# FIGO good practice recommendations on the importance of registry data for monitoring rates and health systems performance in prevention and management of preterm birth

**DOI:** 10.1002/ijgo.13847

**Published:** 2021-09-14

**Authors:** J. Frederik Frøen, Ana Bianchi, Ann‐Beth Moller, Bo Jacobsson, the FIGO Working Group for Preterm Birth, Bo Jacobsson, Joe Leigh Simpson, Jane Norman, William Grobman, Ana Bianchi, Stephen Munjanja, Catalina María Valencia González, Ben Mol, Andrew Shennan

**Affiliations:** ^1^ Global Health Cluster, Division for Health Serrvices Norwegian Institute of Public Health Oslo Norway; ^2^ Perinatal Department Pereira Rossell Hospital, and University Hospital Montevideo Uruguay; ^3^ School of Public Health and Community Medicine Institute of Medicine University of Gothenburg Gothenburg Sweden; ^4^ Department of Obstetrics and Gynecology Institute of Clinical Science Sahlgrenska Academy University of Gothenburg Gothenburg Sweden; ^5^ Department of Obstetrics and Gynecology Region Västra Götaland Sahlgrenska University Hospital Gothenburg Sweden; ^6^ Department of Genetics and Bioinformatics, Domain of Health Data and Digitalization Institute of Public Health Oslo Norway

**Keywords:** health systems strengthening, high‐quality data, preterm birth, prevention, registry

## Abstract

FIGO calls for strengthening of health information systems for reproductive, maternal, newborn, and child health services, co‐designed with users, to ensure the timely accessibility of actionable high‐quality data for all stakeholders engaged in preventing and managing preterm birth consequences. FIGO calls for strengthening of investments and capacity for implementing digital registries and the continuity of reproductive, maternal, newborn, and child health services in line with WHO recommendations, and strengthening of the science of implementation and use of registries—from local quality improvement to big data exploration.

## Synopsis

Effective preterm birth prevention needs strengthened health information systems for maternal and child health services, co‐designed with users, to ensure accountability and actionable high‐quality data for all stakeholders.

## 1 INTRODUCTION

Universal health care coverage, including financial risk protection, is one of the cornerstones of the Global Sustainable Development Goal (SDG) Framework^1^ as well as the Global Strategy for Women's, Children's and Adolescents’ Health (2016–2030).^2^ However, the lack of effective strategies for preventing and managing preterm birth and its consequences is still of significant concern in many low‐ and middle‐income countries (LMICs). Several LMICs struggle to ensure equitable access to use and quality of care even for primary health care for pregnant women and newborns. Despite the evidence of effective preventive strategies, such as antenatal care, many pregnant women do not receive the basic recommended interventions and number of visits.^3^ The *Lancet Global Health* commission on ‘High‐Quality Health Systems in the Sustainable Development Goals Era’ asserted that “Providing health services without guaranteeing a minimum level of quality is ineffective, wasteful, and unethical”.^4^

Timing of birth is paramount, as the risk of neonatal death, severe morbidity, prolonged hospital admissions, and long‐term sequelae increase the lower the gestational duration at preterm birth. Thus, up‐to‐date data of the local and global burden of preterm birth are critical for improving understanding about its epidemiology in order to support and target programs for reducing preterm birth rates over time and inform policies and resource allocation within health systems.

Evidence‐based and data‐driven improvements depend on accessible, accurate, and timely data actionable for pertinent stakeholders—from the local clinic to the global level, from local quality improvement to international science efforts. Health services data are often limited and outdated, and even the most basic data, such as preterm birth rates and mortality rates, are often based on estimations for global reporting purposes due to scarcity of country‐level data. To better understand, prevent, and manage the excessive burden of preterm birth, there is a critical need for more timely data collection and use of health services data as actions should be based on evidence.

*Recommendation*: *FIGO calls for strengthening of health information systems for reproductive*, *maternal*, *newborn*, *and child health services*, *co*‐*designed with users*, *to ensure the timely accessibility of actionable high*‐*quality data for all stakeholders engaged in the prevention and management of preterm birth and its consequences*.

## 2 DIGITAL REGISTRIES ALONG WITH CONTINUITY OF CARE

Every individual counts and should be counted individually. Opportunities and challenges for preventing and managing preterm birth exist along with the complete continuity of public health services and care for the mother–baby dyad—from pre‐pregnancy care to pediatrics and onwards in life. These include preventive programs, such as well‐woman health care, different models for antenatal care, and therapeutic care models, critical to preventing and managing preterm birth and its health outcomes. Along with this continuity, individual‐level longitudinal data emerge and are needed to appropriately observe the quality and continuity of care provision, the prognosis of cohorts, and the denominators of outcome measures. Critically, such real‐life registry data are needed for health technology assessments and post‐implementation evaluations of predictive tests, therapeutic interventions, and preventive care models that may have efficacy in trial settings but uncertain effectiveness when implemented in new contexts at scale.

The ‘WHO Guideline: Recommendations on Digital Interventions for Health System Strengthening’ has summarized current state‐of‐the‐art and recommended established digital health interventions to support adequate reproductive, maternal, newborn, and child health services in LMICs.^5^ Among the digital health interventions recommended by the WHO Guideline Development Group is the digital tracking of clients’ health status and services. Such digital health records that create a database of prospective, longitudinally collected data along with the continuity of care are recommended by the WHO with or without integrated digital health interventions for clinical decision support or targeted client communications (e.g. SMS messaging for reminders of care, test results, individualized health information). The leap from appropriate paper records to advanced health information systems has been seen as an impossible task for many LMICs—and the WHO recommendation comes with numerous contextual implementation considerations. Yet there is a certainty that paper is not the future of the information age. When digital tools are co‐designed appropriately with end‐users, they can limit the burden of data collection and maximize use of the data.^6^ There is a rapidly increasing number of successful implementation experiences of maternal and child health and immunization registries across Latin America, sub‐Saharan Africa, the Middle East, and South‐East Asia. To facilitate implementation, the WHO has published complete suites of ready‐made registry solutions in the reproductive, maternal, newborn, and child health area in the free, open‐source system OpenSRP,^7^ as has the worldwide community of practice of DHIS2, which is used as the health information system in over 70 LMICs.^8^

*Recommendation*: *FIGO calls for strengthening of investments and capacity for implementation of digital registries along with the continuity of reproductive*, *maternal*, *newborn*, *and child health services in line with WHO recommendations*, *and strengthening of the science of implementation and use of registries—from local quality improvement to big data exploration*.

## CONFLICTS OF INTEREST

Bo Jacobbson reports research grants from Swedish Research Council, Norwegian Research Council, March of Dimes, Burroughs Wellcome Fund, and the US National Institute of Health; clinical diagnostic trials on NIPT with Ariosa (completed), Natera (ongoing), Vanadis (completed), and Hologic (ongoing) with expenditures reimbursed per patient; clinical probiotic studies with product provided by FukoPharma (ongoing, no funding), and BioGaia (ongoing; also provided a research grant for the specific study); collaboration in IMPACT study where Roche, Perkin Elmer, and Thermo Fisher provided reagents to PLGF analyses; coordination of scientific conferences and meetings with commercial partners such as NNFM 2015, ESPBC 2016, and a Nordic educational meeting about NIPT and pre‐eclampsia screening. Bo Jacobbson is also Chair of the FIGO Working Group for Preterm Birth and the European Association of Perinatal Medicine special interest group on preterm delivery; steering group member of Genomic Medicine Sweden; chairs the Genomic Medicine Sweden complex diseases group; and is Swedish representative in the Nordic Society of Precision Medicine. All other named authors report no conflicts of interest.

## AUTHOR CONTRIBUTIONS

All authors and the members of the FIGO Working Group for Preterm Birth drafted the concept and idea of the paper. FF wrote the first version of the manuscript. AB, ABM, and BJ revised various versions of the manuscript. All authors and working group members commented on the manuscript and approved the final version.

## MEMBERS OF THE FIGO WORKING GROUP FOR PRE TERM BIRTH, 2018–2021

Bo Jacobsson (Chair), Joe Leigh Simpson, Jane Norman, William Grobman, Ana Bianchi, Stephen Munjanja, Catalina María Valencia González, Ben W. Mol, Andrew Shennan
